# Paradigm shifts in prostate biopsy strategies in the era of precision medicine

**DOI:** 10.3389/fsurg.2026.1928043

**Published:** 2026-10-09

**Authors:** Chenhui Tang, Zhen Liang, Weigang Yan

**Affiliations:** Department of Urology, Peking Union Medical College Hospital, Chinese Academy of Medical Sciences & Peking Union Medical College, Beijing, China

**Keywords:** artificial intelligence, biopsy, precision medicine, prostate, prostate cancer

## Abstract

The escalating global burden of prostate cancer necessitates continuous refinement of diagnostic strategies. Although prostate biopsy remains the gold standard for diagnosis, key procedural aspects, including biopsy template, guidance methods, and biopsy route, remain subjects of ongoing debate. Current approaches have yet to achieve the dual goals of high sensitivity and high specificity in diagnostic performance. In recent years, ongoing advancements in related fields have generated substantial clinical evidence, offering further insights into optimizing biopsy strategies. This review provides a synthesized framework for navigating the paradigm shift from traditional, standardized biopsy toward personalized, precision-based approaches. We not only trace the evolution of biopsy techniques but also offer a comprehensive, in-depth exploration of how patient-specific factors, such as prostate volume, prostate-specific antigen density, and Prostate Imaging Reporting and Data System scores, can inform individualized biopsy decisions, thereby providing practical clinical guidance. In addition to critically evaluating conventional biopsy paradigms, we systematically examine emerging biopsy strategies and synthesize high-quality studies at the forefront of this field. Finally, against the backdrop of the artificial intelligence era, we highlight the transformative potential of artificial intelligence and robotic platforms across the diagnosis. By integrating current development and future directions, this review offers a structured framework for personalized biopsy decision-making, ultimately aiming to achieve both high sensitivity and high specificity in individualized prostate cancer diagnosis.

## 1 Introduction

Prostate cancer (PCa) remains the most commonly diagnosed malignant tumor among men in 112 countries, with global incidence projected to rise from approximately 1.4 million new cases in 2020 to 2.9 million by 2040. This anticipated increase is largely attributable to demographic shifts and improvements in life expectancy, which collectively contribute to a growing population of individuals at risk (1). Against this backdrop, the need for precise risk stratification and individualized screening strategies has become increasingly urgent. Specifically, those with intermediate- or high-risk disease require timely and accurate diagnosis to prevent progression to metastatic or advanced stages, while low-risk patients should be spared unnecessary diagnostic interventions to avoid overdiagnosis and overtreatment.

Building upon this clinical imperative, major international guidelines have established a structured diagnostic pathway for prostate cancer. The initial evaluation typically includes digital rectal examination and serum prostate-specific antigen (PSA) testing, followed by further characterization through magnetic resonance imaging (MRI). When imaging findings suggest the presence of malignancy, prostate biopsy is indicated as the definitive diagnostic procedure. Among available diagnostic modalities, prostate biopsy occupies a unique position as the gold standard for definitive diagnosis, as it enables direct histopathological assessment of prostatic tissue. The clinical significance of biopsy findings is systematically interpreted using the Gleason Grade (GG) system proposed by the International Society of Urological Pathology, which stratifies patients based on prognostic implications: GG 1 is classified as clinically insignificant PCa (cisPCa), whereas GG 2 through GG 5 represent clinically significant PCa (csPCa). This histopathological classification serves as the foundation for subsequent treatment decisions and prognosis estimation.

Prostate biopsy is central to the diagnostic pathway, and biopsy techniques have evolved rapidly. Therefore, a comprehensive synthesis of current high-quality evidence, including randomized controlled trials (RCTs), is warranted. This article presents a review of advancements in prostate biopsy strategies. We begin by delineating the historical evolution of biopsy techniques, tracing the paradigm shift from the conventional systematic method to the contemporary integration of targeted and systematic approaches. Subsequently, we evaluate technological innovations in biopsy guidance methods, with particular emphasis on the diagnostic accuracy and clinical applicability of multiparametric MRI (mpMRI), micro-ultrasound (micro-US) techniques, and Prostate-Specific Membrane Antigen Positron Emission Tomography/Computed Tomography (PSMA-PET/CT). The ongoing debate regarding optimal biopsy routes is then critically examined, synthesizing current evidence that compares the transperineal and transrectal approaches while considering factors that guide individualized selection. Finally, we explore emerging roles for artificial intelligence (AI) and robotic-assisted platforms in the biopsy workflow and propose directions for future research.

This review aspires to navigate the ongoing paradigm shift in prostate biopsy by addressing its central challenge: the pursuit of a personalized optimal balance between diagnostic precision and minimal procedural invasiveness. We hope to bridge the gap between technological evolution and clinical decision-making in prostate cancer diagnosis. By providing a structured synthesis of past paradigms, present evidence, and future possibilities, we aim to equip clinicians with the insights necessary for personalized patient care, while simultaneously laying a cornerstone for future scholarly inquiry that will continue to refine the diagnostic pathway and improve outcomes for men at risk.

## 2 Methods

This article is a narrative review of recent advances in prostate biopsy strategies. The literature was identified through searches of PubMed, Embase and Web of Science, covering the period from 05/2021 to 05/2026. The principal search terms included combinations of keywords related to prostate cancer and biopsy, including “prostate cancer”, “prostate biopsy”, “systematic biopsy”, “targeted biopsy”, “combined biopsy”, “perilesional biopsy”, “unilateral systematic biopsy”, “saturation biopsy”, “transperineal biopsy”, “transrectal biopsy”, “multiparametric MRI”, “micro-ultrasound”, “PSMA-PET/CT”, “artificial intelligence” and “robotic-assisted biopsy”. Reference lists of relevant articles and guidelines were also screened to identify additional studies of clinical importance.

Literature selection was based on clinical relevance, methodological quality, novelty, and applicability to contemporary prostate biopsy practice. RCTs were prioritized when they used appropriate randomization, clinically relevant endpoints, adequate sample sizes, complete follow-up, appropriate controls, and contemporary biopsy protocols. Systematic reviews and meta-analyses were prioritized when they used comprehensive searches, explicit eligibility criteria, appropriate risk-of-bias and heterogeneity assessments, and transparent reporting of evidence certainty. Guidelines were considered when based on systematic evidence review, graded recommendations, and multidisciplinary consensus. Prospective multicenter studies were preferred for evaluating emerging techniques, particularly when they used standardized protocols, prespecified endpoints, and external validation. When higher-level evidence was unavailable, well-designed prospective or retrospective observational studies were included, with emphasis on clearly defined populations and outcomes, appropriate adjustment for confounding, and adequate follow-up. Studies with the most rigorous design, lowest apparent risk of bias, and greatest clinical applicability were emphasized, while landmark studies were retained for historical context. This approach provided a balanced narrative synthesis without formal evidence grading or quantitative pooling.

## 3 Evolution of traditional biopsy strategies

Systematic biopsy (SBx) and targeted biopsy (TBx) have a long and established history. In 1989, Hodge et al. first proposed the systematic sextant biopsy technique, performing transrectal ultrasound-guided core biopsies of the prostate (2). Following the introduction of this six-core approach, subsequent investigations have driven continuous refinements in biopsy templates, sampling numbers, and procedural techniques, leading to the exploration of various protocols such as five-region transrectal biopsy, as well as 10-core, 11-core, 12-core, and TBx strategies. Currently, the 12-core systematic biopsy and TBx are the most widely adopted approaches in clinical practice. This section summarizes current research progress on the most frequently employed biopsy strategies, aiming to provide evidence-based recommendations for the personalized selection of biopsy protocols through comparative analysis and synthesis of findings across studies.

### 3.1 Systematic biopsy and targeted biopsy

SBx has long been the cornerstone of PCa diagnosis due to its standardization, simple equipment requirements, and ability to detect MRI-invisible tumors (Figure 1A). However, its non-targeted, grid-based approach assumes uniform cancer distribution, leading to blind sampling, diagnostic omission, low detection in specific regions, overdiagnosis, and increased complication risk from higher core numbers. In contrast, TBx leverages MRI to enable targeted sampling. By fusing real-time ultrasound with pre-acquired MRI, TBx selectively samples suspicious regions, reducing diagnostic omission and avoiding overdiagnosis (Figure 1B).

**Figure 1:**
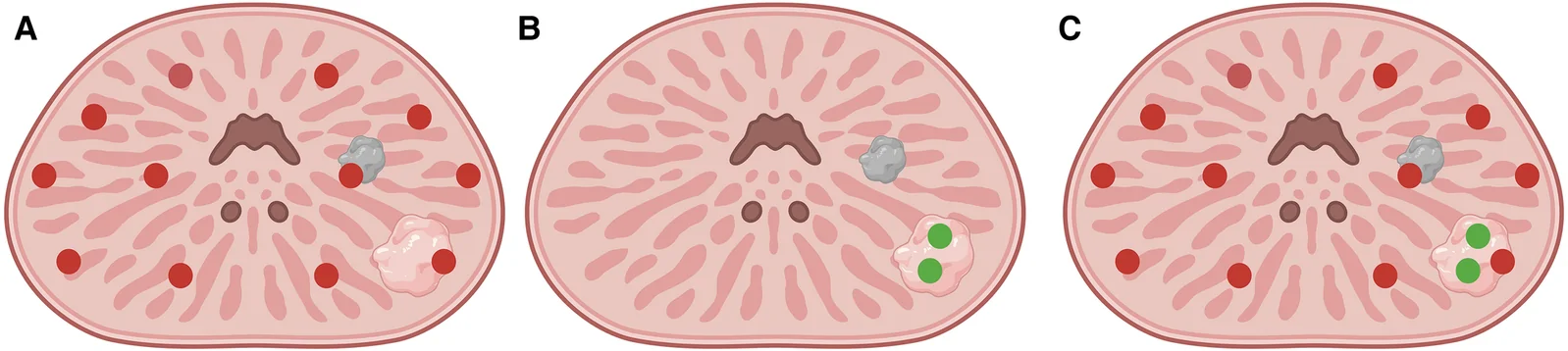
Sketch maps of systematic biopsy **(A)**, targeted biopsy **(B)** and combined biopsy **(C)**. The red dots represent systematic biopsy sites, and the green dots represent targeted biopsy sites. The pink mass represents a lesion visible in MRI, and the grey mass represents a lesion invisible in MRI.

Extensive studies have shown that when a visible lesion is present on mpMRI, a targeted biopsy strategy may enhance the detection of csPCa. Patel et al.'s large-scale retrospective study reported that TBx demonstrated a higher detection rate for csPCa compared to SBx, at 27.9% versus 23.3% (3). In recent years, further RCTs have compared biopsy strategies for PCa diagnosis. The VISION study, an individual patient data meta-analysis of the PRECISION (4) and PRECISE (5) RCTs, showed that an MRI-based strategy incorporating biopsy avoidance may not substantially increase the risk of missed or delayed detection of csPCa over the short term, with a higher detection rate for csPCa (36.3% vs. 27.6%) and a lower rate for cisPCa (9.6% vs. 21.9%). Moreover, 32.2% of men in the MRI arm avoided initial biopsy due to non-suspicious MRI findings, and had a significantly higher proportion of biopsy cores positive for cancer (48.8% vs. 20.4%), indicating greater diagnostic efficiency with fewer cores (6). The 2-year follow-up of the PRECISE trial demonstrated no significant difference in the delayed diagnosis rate of csPCa between the two study arms. Among men with negative MRI who avoided initial biopsy, only 5% were subsequently diagnosed with Gleason 3 + 4 diseases at two years, with no cases of higher-grade cancer detected (7). These findings suggest that an MRI-based strategy with biopsy avoidance does not lead to substantial missed diagnoses or delayed detection of csPCa. The recent GOTEBORG-2 randomized trial further corroborated these results, reporting that the TBx strategy for men with PSA ≥3 ng/mL led to a 50% reduction in the detection of cisPCa compared with a strategy of SBx (0.6% vs. 1.2%) (8). This reduction in overdiagnosis was achieved without a statistically significant difference in the detection of csPCa between the groups.

However, the PROBASE RCT in men aged 45–50 years showed that SBx identified 94% of csPCa, while TBx detected only 74%, and further analysis showed SBx missed fewer csPCa cases than TBx (16% vs. 33%) (9). The results show that a strategy relying solely on MRI-targeted biopsy of individual lesions can miss a considerable proportion of csPCa, despite their visibility on imaging. This may be due to smaller prostate volumes in younger men, tumor location, and MRI interpretation challenges, warranting the necessity of SBx in this population. Considering the limitations of MRI, such as the invisibility of lesions or atypical imaging findings, SBx still has irreplaceable value in comprehensively evaluating prostate cancer and avoiding missed diagnosis of csPCa.

Both TBx and SBx exhibit distinct merits, raising the question of how to formulate an optimal strategic selection. Conventional clinical indicators provide an important reference for such decision-making. First, prostate volume serves as a critical parameter. Evidence has shown that a larger discrepancy between prostate volumes measured by ultrasound and MRI in the initial evaluation correlates with diminished diagnostic superiority of TBx (10). Specifically, when the absolute volume difference exceeded 5 mL, TBx showed a significant decrease in the detection of csPCa, relative advantages over SBx, and the ability to detect missed cases. Secondly, PSA density (PSAD) constitutes another key index. For patients with a PSAD ≥0.2 ng/mL/cm^3^, it may be reasonable to omit SBx and perform TBx exclusively for MRI-visible lesions (11). What's more, Prostate Imaging Reporting and Data System (PI-RADS) scores exert a substantial influence on biopsy strategy. Given that TBx demonstrates higher detection rates of csPCa in patients with higher PI-RADS scores (3), some studies suggest that in cases with a PI-RADS score of 5, TBx alone may be sufficient, whereas for those with scores of 3 or 4, a combined TBx and SBx approach may be necessary to ensure diagnostic reliability (12).

### 3.2 Combined biopsy: the synergistic integration of systematic and targeted approaches

Given that both biopsy strategies possess distinct advantages and limitations, the combined biopsy (CBx), which combines TBx and SBx, has higher clinical practical significance (Figure 1C). This is supported by the MULTIPROS trial, which demonstrated that among patients with PI-RADS ≥3, the combined biopsy strategy yielded a higher detection rate of csPCa, indicating a statistically significant superiority over either method alone (13). Further investigations have explored the performance of these strategies across different patient subgroups. Take patients in active surveillance (AS) for example, a retrospective study confirmed that the use of CBx for initial or confirmatory diagnosis facilitates more accurate selection of candidates for AS, significantly reducing rates of pathological upgrading and AS disqualification compared to SBx (19% vs. 59%) (14). What's more, in a prospective single-center study, Liu et al. reported that among biopsy-naïve patients, CBx detected more csPCa cases than SBx alone (49.1% vs. 43.0%). This advantage was particularly pronounced in patients with a PSAD > 0.15 ng/mL and those with PI-RADS scores of 4–5 (15). Collectively, these studies underscore the growing evidence that CBx not only improves csPCa detection overall but also offers particular benefits in specific clinical contexts.

CBx remains the recommended strategy in most high-quality studies. However, growing concerns about overtreatment require reassessment of its net clinical benefit. Compared with SBx alone or TBx alone, combined biopsy significantly reduces the rate of pathological upgrading at radical prostatectomy but increases the risk of pathological downgrading. As downgrading is also considered a clinically meaningful harm due to its potential to lead to unnecessary surgical intervention, the overall benefit of combined biopsy may be partially or fully offset (16). On the other hand, since SBx has long been criticized for its contribution to overdiagnosis, combined biopsy, by virtue of incorporating a systematic component, naturally faces the same challenge. At the individual lesion level, TBx and SBx demonstrated a 95% overlap in detecting the primary tumor pattern. SBx provided additional diagnostic information beyond the targeted area in only 5% of cases; notably, the vast majority of these additionally detected lesions were low-volume, exhibited low aggressiveness, and carried exceptionally low clinical risk (17). Consequently, reseachers take efforts to optimize the standard 12-core SBx protocol in the context of CBx strategies. Deng et al. revealed that a novel CBx scheme (TB + 6SB) demonstrated non-inferior detection of csPCa compared to the classical 12-core protocol (54.3% vs. 54.8%), while significantly improving patient safety by reducing procedural discomfort and post-biopsy pain (18). Based on these findings, this study suggests that reducing SBx cores while maintaining accuracy and enhancing safety represents a key direction for future improvements in CBx, particularly for biopsy-naïve patients with solitary MRI-suspicious lesions.

## 4 Extension of traditional biopsy strategies

Despite the established role of SBx and TBx in prostate cancer diagnosis, ongoing concerns regarding diagnostic yield, sampling error, and procedural morbidity have driven continued refinements in biopsy strategy. A range of modified approaches based on TBx have subsequently emerged, aiming to balance detection accuracy with patient burden. Generally, perilesional biopsy (PBx) focuses on sampling around MRI-visible lesions, while unilateral systematic biopsy (uni-SBx) limits sampling to one lobe in selected patients, and saturation methods employ extensive repeat sampling for targeted lesions (Figure 2). This section reviews the current evidence on these alternative biopsy strategies.

**Figure 2:**
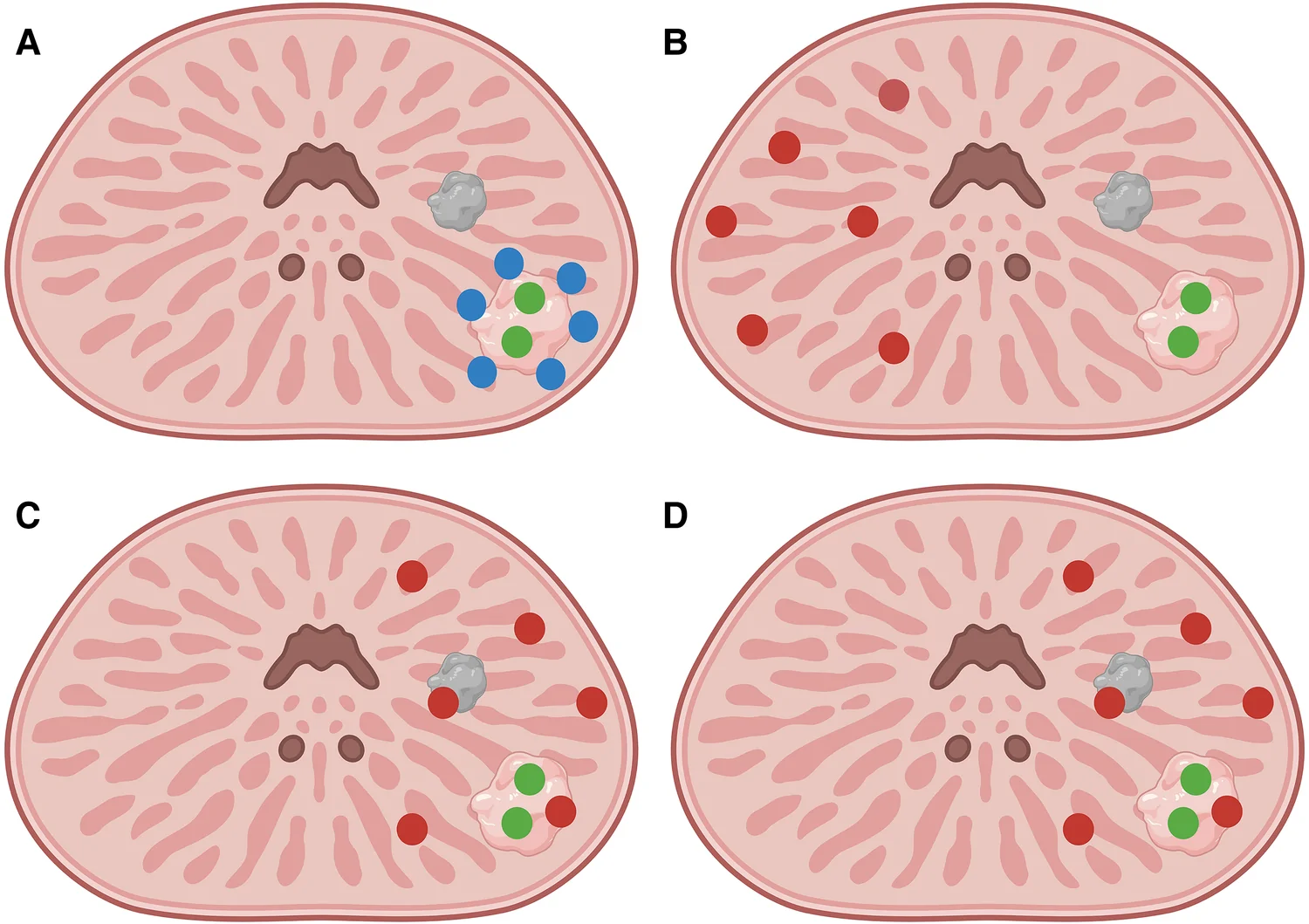
Extension of targeted biopsy. **(A)** Perilesional biopsy. **(B)** Contralateral systematic biopsy + targeted biopsy. **(C)** Ipsilateral systematic biopsy + targeted biopsy. **(D)** Targeted saturation biopsy. The blue dots represent extra perilesional biopsy sites around the targeted biopsy sites, the red dots represent systematic biopsy sites, and the green dots represent targeted biopsy sites **(A–C)** and targeted saturation biopsy sites **(D)**. The pink mass represents a lesion visible in MRI, and the grey mass represents a lesion invisible in MRI.

### 4.1 Perilesional biopsy

The latest EAU guidelines suggest targeted biopsy combined with perilesional sampling as an alternative sampling strategy for selected patients with MRI-positive lesions (PI-RADS ≥4), reflecting a shift toward more lesion-focused biopsy strategies. PBx involves additional sampling around the suspicious lesion on mpMRI without significantly reducing the total number of cores, but rather redistributing them: concentrating on the lesion while omitting random sampling in negative regions (Figure 2A). This strategy aims to avoid missed diagnoses while minimizing overdiagnosis and overtreatment.

In Fakunle et al.'s study, approximately 25% of lesions were identified in regions contiguous to the primary target during TBx. This finding suggests that more extensive sampling at the periphery of the target may be warranted during targeted biopsy to enhance overall detection sensitivity (17). A meta-analysis incorporating 21 studies compared the diagnostic efficacy of CBx versus PBx for csPCa. The results showed no significant difference in the overall detection rate of csPCa between the two methods (46.1% vs. 44.2%). However, CBx demonstrated a significantly higher detection rate for cisPCa (OR = 1.16), suggesting that PBx in early diagnosis could help avoid unnecessary systematic biopsies while reducing the overdetection of indolent tumors (19). The recently published MIRAGE trial, a prospective, observational, multicenter study by Baboudjian et al., found similar conclusions, with an increase in the detection of csPCa by 3.6%, at the cost of a 1% increase in the detection of cisPCa (20). What's more, the latest PERI-PRO RCT by Deng et al. also demonstrated the non-inferiority of the perilesional biopsy strategy for the detection of csPCa. They found the PBx group showed significantly lower rates of overall complications (62% vs. 74%), particularly bleeding-related adverse events and rectal pain (21). It is worth mentioning that similar conclusions regarding the non-inferiority of this approach were previously reported by Noujeim et al., further supporting its potential as an alternative to SBx in selected patients (22).

Although PBx improved the detection rate of csPCa compared with TBx alone (30% vs. 19%), it remained insufficient to replace CBx, which achieved a significantly higher detection rate (49% vs. 30%). In confirmatory biopsies during AS, neither TBx nor PBx reliably rules out csPCa (23). Given that pathological upgrading in AS patients directly dictates therapeutic reclassification, it's advocated for a CBx approach incorporating both targeted and extensive systematic sampling. Generally, existing evidence suggests that PBx can achieve diagnostic accuracy for csPCa comparable to that of CBx in select populations, while offering a more favorable safety profile and reducing the overdiagnosis of indolent tumors. This underscores the need for optimized patient selection to enable individualized biopsy strategies.

### 4.2 Unilateral systematic biopsy

Uni-SBx is designed to reduce unnecessary sampling and potential overdiagnosis by omitting systematic cores from one side, guided by the location of an MRI-visible lesion. This approach can be considered a specific form of PBx or an adaptation of CBx, and is typically performed in conjunction with targeted cores. While the concept encompasses both ipsilateral (ips-SBx) and contralateral (con-SBx) systematic sampling (Figures 2B, C), the ipsilateral variant is more widely adopted and clinically accepted. A multicenter study found that the contribution of con-SBx was extremely limited, with only 1.5% of patients having csPCa detected exclusively by this approach (24). A recent meta-analysis further corroborated these findings: in terms of csPCa detection, ips-SBx combined with TBx demonstrated a comparable detection rate to con-TBx (RR 0.98), and significantly fewer cisPCa cases were detected with ips-SBx plus TBx compared with CBx (RR 0.86) (25). When compared with CBx, Wu et al. also demonstrated that ips-SBx plus TBx didn't show statistically significant inferiority in detecting csPCa (RR 0.95) and significantly reduced the detection rate of cisPCa (RR 0.87) (26). These findings suggest that ips-SBx combined with TBx may represent a potential compromise, preserving csPCa detection while reducing overdiagnosis of indolent disease.

Uni-SBx represents an extension of the perilesional biopsy approach, grounded in the traditional template of systematic sextant biopsy. Notably, an alternative direction of extension of uni-SBx is based on the zonal anatomy of the prostate. Specifically, for patients with MRI-visible lesions confined to the peripheral zone, omitting systematic sampling of the transition zone during fusion biopsy has a negligible impact on the detection rate of csPCa (47% vs. 48%) (27). Besides relying on left-right symmetry, it may be possible in the future to further extend uni-SBx by leveraging other three-dimensional structural characteristics of the prostate, such as zonal anatomy (transition vs. peripheral zone), anteroposterior gradient, or apical-basal distribution.

### 4.3 Targeted saturation biopsy

Saturation biopsy primarily comprises two strategies: saturation systematic biopsy and targeted saturation biopsy (TSx). Given the very low risk of subsequent csPCa following a negative biopsy and/or a negative MRI, the clinical utility of saturation systematic biopsy remains uncertain within the current MRI-driven diagnostic pathway. Actually, TSx is more frequently adopted in current clinical practice, which entails intensive, protocol-driven sampling of a targeted lesion to optimize the detection of csPCa and more precisely delineate its spatial extent (Figure 2D).

Early studies evaluating TSx demonstrated its diagnostic potential, and subgroup analyses further revealed that its superiority in detecting csPCa was predominantly observed in patients with PI-RADS 4 lesions (28). More recently, Saner et al.'s RCT compared conventional TBx (four cores per lesion) with TSx (nine cores per lesion). The results showed no significant difference in csPCa detection rates between the two approaches (TSx vs. TBx: 100% vs. 91.7%) (29). Despite taking more cores from the lesion, TSx does not appear to increase the detection of cisPCa. A meta-analysis by Wu et al. demonstrated that, compared with CBx, TSx significantly decreased the detection rate of cisPCa (RR 0.76), while achieving a comparable csPCa detection rate (26). Supporting this notion, a prospective study by Morote et al. reported similar results, with TSx yielding a higher csPCa detection rate (71.9% vs. 47.9%) and a lower cisPCa detection rate (11.1% vs. 18.8%) compared to TBx (30). These findings suggest that TSx strategies may help mitigate overdiagnosis without compromising the detection of csPCa.

As previously discussed, CBx represents a trade-off between SBx and TBx. Within this context, the question arises as to whether TSx still holds clinical value. The latest meta-analysis demonstrated that, compared with CBx, neither saturation SBx plus TBx nor SBx plus saturation TBx showed a statistically significant difference in csPCa detection rates (26). Furthermore, an RCT revealed that although TSx achieved a csPCa detection rate comparable to that of CBx (44.1% vs. 40.7%), it yielded a higher positive core rate (32.7% vs. 18.3%) while requiring fewer biopsy cores (31). These findings suggest that TSx strategies may optimize procedural efficiency and reduce unnecessary tissue sampling without compromising diagnostic accuracy. Nevertheless, the widespread adoption of TSx in clinical practice still requires validation through multicenter studies. Future efforts should focus on standardizing the number of cores for TSx and conducting clinical studies to further investigate the necessity of performing part or all of SBx during TSx procedures.

## 5 Advancements in biopsy guidance methods

Precision in image guidance is fundamental to ensuring both the accuracy and safety of prostate biopsy procedures. With ongoing advances in medical imaging and interventional devices, guidance methods for prostate biopsy have undergone continuous evolution from conventional techniques to innovative approaches. This section provides an in-depth review of currently available guidance modalities in clinical practice. We first examine the development and clinical utility of conventional approaches, followed by a discussion of the principles and advantages of emerging technologies, including micro-US and PSMA-PET/CT, aiming to inform clinical decision-making in selecting appropriate guidance strategies.

### 5.1 Conventional guidance approaches

After confirming the target biopsy site, ensuring that the biopsy needle accurately reaches the lesion under image guidance is a critical challenge in prostate cancer diagnosis. Image-guided targeted biopsy plays an indispensable role in the detection of prostate cancer. Currently, the primary approaches for targeted biopsy include three modalities: multiparametric MRI-guided in-bore targeted biopsy (in-bore TBx), MRI and transrectal ultrasound fusion targeted biopsy (fus-TBx), and cognitive fusion-targeted prostate biopsy (cog-TBx). In-bore TBx involves fusing previously acquired MRI data showing suspicious lesions with real-time MRI scans to guide needle placement during biopsy. As for fus-TBx, the prostate contour and suspicious targets are delineated on MRI images using fusion software prior to biopsy. During the procedure, these MRI images are registered, fused, and aligned with real-time Transrectal Ultrasound (TRUS) images, enabling precise needle targeting of suspicious lesions under TRUS guidance. Different from the previous two methods, cog-TBx relies on the operator's integration of MRI-identified lesion locations with real-time TRUS images during the procedure, thereby directing the biopsy process based on subjective alignment.

Early studies exhibited significant heterogeneity, complicating direct comparisons of the three targeted biopsy methods. One important reason is that the diagnostic efficiency of targeted biopsy is highly dependent on MRI image quality. Overall, current evidence indicates no significant differences among the three guidance modalities for detecting csPCa. A recent meta-analysis encompassing 4928 patients reported comparable detection rates for csPCa (in-bore TBx: 0.47; fus-TBx: 0.39; cog-TBx: 0.37) and cisPCa (in-bore TBx: 0.18; fus-TBx: 0.17; cog-TBx: 0.12) (32). However, the comparative efficacy of these techniques may be influenced by tumor location, as fus-TBx appears superior for anterior and transition zone tumors, whereas cog-TBx is more effective for basal lesions, suggesting potential complementarity (33). Patient baseline characteristics also play a significant role in diagnostic outcomes. Among biopsy-naïve patients with PSA 2–10 ng/mL, Izadpanahi et al.'s RCT demonstrated that fus-TBx was associated with significantly improved overall prostate cancer detection (44.4% vs. 31.0%) and csPCa detection (33.3% vs. 19.0%) relative to cog-TBx (34).

In-bore TBx may offer superior detection rates for both overall PCa and csPCa compared with other biopsy approaches (33). However, Schouten et al. raised concerns that this technique could miss apical or dorsolateral lesions (35), a finding later challenged by Schaudinn et al., who found no location-based differences but identified lesion size as a significant predictor of csPCa detection (36). Sattin et al. further confirmed that csPCa detection was significantly influenced by PI-RADS category (PI-RADS ≥4) and lesion size (>10 mm) (37). It is worth mentioning that in patients with PI-RADS 4–5 lesions, Prince et al. demonstrated that in-bore TBx achieved significantly higher target-specific cancer detection than fus-TBx (OR = 2.28) (38). Despite these advantages, Sattin et al. reported that 66.1% of patients with GG1 on biopsy experienced upgrading at radical prostatectomy (37). Generally, in-bore TBx remains a time-consuming, costly, and complicated procedure, and high-quality prospective studies confirming its diagnostic accuracy for csPCa are still lacking, limiting its widespread clinical adoption.

### 5.2 Novel guidance approaches

Beyond conventional image fusion guidance, a range of novel targeted biopsy techniques are increasingly being integrated into prostate cancer diagnostic pathways. The prospective single-center CRAFT study by Fleville et al., which employed biparametric MRI (bpMRI) in biopsy-naïve men, reported that targeted biopsy detected prostate cancer and csPCa in 86.2% and 76.5% of cases, respectively, compared with 78.7% and 56.3% using fusion biopsy (39). A study by Schieda et al. further demonstrated that bpMRI and mpMRI-guided TRUS-targeted biopsies yielded equivalent positive predictive values for csPCa, both at 34%, suggesting that the omission of dynamic contrast-enhanced sequences does not compromise diagnostic performance (40). From a technical perspective, the integration of advanced electromagnetic needle tracking systems into conventional MRI-TRUS fusion platforms has enabled real-time visualization and tracking of biopsy needles. This innovation enhances the precision of targeting MRI-visible lesions, contributing to sustained high csPCa detection rates while improving patient tolerance (41).

Micro-US has also emerged as a promising modality for targeted biopsy guidance. Early prospective studies demonstrated that, even in the MRI era, micro-US provides complementary information to mpMRI-guided TBx, whether via the transrectal or transperineal route, facilitating the detection of csPCa (42–44). Furthermore, at the level of lesions, approximately 67% of lesions identified on MRI are also visualized in real time on micro-US, enabling its use as a direct guidance tool for immediate targeted biopsy (45).

The recently published OPTIMUM RCT compared three diagnostic strategies: micro-US-guided biopsy, fus-TBx, and combined micro-US plus fus-TBx. The csPCa detection rates were 47.1% in the micro-US group and 42.6% in the fus-TBx, confirming the non-inferiority of micro-US. Moreover, the combined approach also demonstrated non-inferiority, indicating that micro-US can be effectively integrated with existing fusion platforms without compromising diagnostic yield (46). Researchers further compared cognitively targeted micro-US biopsy with fus-TBx for detecting csPCa, finding that the former achieved a higher overall detection rate (45% vs. 34%) (47). Collectively, these advances underscore a paradigm shift toward more flexible, precise, and patient-adaptive biopsy guiding methods.

PSMA-PET/CT has also demonstrated significant advantages in TBx. Available meta-analyses have shown that PSMA-PET/CT TBx exhibits high sensitivity but low specificity for detecting csPCa (48, 49). A single-center RCT further confirmed that ^68^Ga-PSMA-11 PET/CT TBx achieved a csPCa detection rate of 40.0%, significantly higher than the 25.0% rate with SBx; this advantage was more pronounced in patients with PSMA-positive lesions or PSA 4.0–20.0 ng/mL (50). However, when combined with conventional TBx, PSMA-PET/CT TBx demonstrated high diagnostic performance, with a sensitivity of 0.91 and specificity of 0.64 (48). A recent multicenter RCT reported that combining PSMA-PET/CT TBx with SBx yielded a csPCa detection rate of 67.0%, which was superior to either approach alone (51). Furthermore, dual-tracer (GRPR and PSMA) PET/CT-guided biopsy showed notable performance, with a detection rate of 69.77% and the ability to avoid more than half of unnecessary biopsies. When combined with SBx, the detection rate increased to 77.36% without missing csPCa, highlighting the potential of multi-modal image-guided biopsy (52).

## 6 Evolution on biopsy routes: transperineal versus transrectal approach

Prostate biopsy modalities encompass the transrectal approach (transrectal prostate biopsy, TRBx) and the transperineal approach (transperineal prostate biopsy, TPBx), each manifesting distinct advantages and limitations across procedural techniques, cancer detection efficacy, and infectious complication risks. A growing body of evidence, including four recent large-scale RCTs (ProBE-PC (53), PERFECT (54), PREVENT (55), TRANSLATE (56), detailed in Table 1), has shifted clinical consensus toward TPBx. These trials substantially strengthened the evidence base for TPBx, primarily attributable to its comparable csPCa detection rate and substantially reduced infectious complication incidence. Nevertheless, the clinical debate regarding the optimal biopsy approach remains far from conclusive. Collectively, these landmark trials, in conjunction with complementary studies investigating patient-reported quality of life and postoperative recovery outcomes, have refined the existing evidence base yet failed to fully resolve the prevailing clinical equipoise. This section undertakes a critical synthesis of the latest trial data to systematically compare the two biopsy strategies, with the objective of not only delineating their core differences but also establishing a conceptual framework for evaluating clinical trade-offs in contemporary practice and guiding future investigative endeavors.

**Table 1:** Rapid review of existing RCTs on biopsy route selection.

Name of trial	Participants	Sample size	Biopsy protocol	Primary endpoint	csPCa detection rate (TRBx vs. TPBx)	Infectious complication rate (TRBx vs. TPBx)^b^
ProBE-PC (53, 57)	Biopsy-naïve or previous negative biopsy	398 TP vs. 384 TR; 367 TP vs. 351 TR^a^	CBx, or SBx only if MRI negative/not performed	csPCa detection; 30-day composite infectious complication rate	47.1% vs. 43.2% (OR 1.09, 95% CI 0.88–1.55)	2.6% vs. 2.7% (OR 1.06, 95% CI 0.43–2.65)
PERFECT (54)	Biopsy-naïve; PI-RADS 4–5	122 TP vs. 128 TR	TBx	csPCa detection rate	47.2% vs. 54.2% (*p* = 0.6235)	0.8% vs. 0.0% (NS)
PREVENT (55)	Biopsy-naïve; PIRADS 3–5	287 TP vs. 280 TR	CBx or SBx only if MRI negative/not performed	7-day composite infectious complication rate	50.4% vs. 52.6% (adjusted difference 2.0%; 95% CI −6.0%∼10%)	1.4% vs. 0.0% (difference −1.4%, 95% CI −3.2%∼0.3%)
TRANSLATE (56)	Biopsy-naïve	547 TP vs. 540 TR	CBx or SBx only if MRI negative/not performed	csPCa detection rate	54.4% vs. 60.1% (OR 1.32; 95% CI 1.03–1.70)	1.6% vs. 0.4% (OR 0.22; 95% CI 0.05–1.01)

a Values represent analyzed sets for the co-primary endpoints: 782 for csPCa (randomized 840) and 718 for infection (randomized 763).

b Definitions of infectious complications varied across trials; direct cross-trial comparisons should be made with caution.

### 6.1 Diagnostic accuracy

Evidence derived from large-scale RCTs demonstrates no statistically significant difference between the two biopsy modalities with respect to csPCa detection (Table 1). Nevertheless, this conclusion was further clarified and contextualized through additional subgroup analyses, which yielded novel findings and offered distinct interpretive perspectives. For example, the ProBE-PC trial reported csPCa detection rates of 59% for TRBx versus 62% for TPBx in biopsy-naïve patients [53; although TRBx exhibited a slightly higher csPCa detection rate in overall patients (47.1% vs. 43.2%). Furthermore, csPCa detection efficacy is influenced not only by prior biopsy status but also by PI-RADS scores. Subgroup analyses have suggested a higher csPCa detection rate with TPBx among patients with PI-RADS 4 lesions, whereas no analogous benefit was observed in the PI-RADS 3 or PI-RADS 5 subgroups (58, 59).

The ability of the two biopsy techniques to detect PCa across distinct prostatic anatomical regions differs substantially. Schieda et al. demonstrated that TPBx yields superior diagnostic sensitivity for anterior lesions (29% vs. 16%) and PI-RADS 4 lesions (51% vs. 30%) (60). Similar results were found in an AS cohort, as Nalavenkata and colleagues observed that TPBx exhibited greater efficacy in sampling anterior tumors, while TRBx showed a predilection for detecting lesions located in the posterior prostate (61). The PERFECT trial further reinforced this spatial complementarity, reporting that TPBx identified fewer posterior tumors (44.3% vs. 59.0%) and more anterior tumors (40.6% vs. 26.5%) compared to TRBx (54). Subsequent meta-analyses from two additional studies consistently affirmed TPBx's heightened sensitivity for anterior and apical lesions (59, 62).

### 6.2 Infectious complication rate

Existing RCTs generally indicate that TPBx is non-inferior to TRBx in terms of the incidence of infectious complications (Table 1). Notably, overall infection rates associated with both approaches are relatively low, and the occurrence of severe life-threatening events such as sepsis is exceedingly rare. Indeed, alongside advances in clinical practice and perioperative management, the overall incidence of biopsy-related infections has declined substantially in recent years.

Although current guidelines uniformly recommend that routine antibiotic prophylaxis is not required prior to TPBx, the necessity of antimicrobial prophylaxis for TPBx has remained a topic of considerable clinical interest. Collectively, available evidence demonstrates no significant differences in the rates of infection, fever, sepsis, or hospital readmission between patients receiving antibiotic prophylaxis and those undergoing antibiotic-free TPBx (63). These findings are primarily generalizable to patients at low infectious risk. However, individuals at high risk of infectious complications or those with active infections require individualized assessment, as the favorable safety profile of antibiotic-free TPBx may not be applicable to such high-risk subgroups.

### 6.3 Non-infectious complications, patient experience and cost-effectiveness

Available RCTs have consistently found no significant differences between TPBx and TRBx prostate biopsy in terms of overall patient comfort and non-infectious complication rates. However, the PREVENT trial offered a more nuanced perspective, noting that while patients undergoing TP biopsy under local anesthesia may report more severe immediate post-procedural pain, which is assessed immediately after biopsy using a 0-10 numerical rating scale, this discomfort is typically transient and resolves within seven days (55). Specifically, the adjusted mean difference in biopsy pain was 0.6 on the 0–10 scale immediately post-procedure, whereas at 7 days the difference in pain scores was 0.3 and the proportion of patients reporting pain >3 was similar between groups (6.6% vs. 6.0%). This finding underscores that the immediate pain associated with TP biopsy, though possibly more intense, is self-limiting and short-term in nature. Emerging evidence highlights the influence of sociodemographic factors on pain perception. A multivariable analysis identified younger age, Black or Latino ethnicity, and a higher number of MRI-visible lesions as independent predictors of increased pain levels (64). These findings suggest that patient sociodemographic characteristics should be integrated into pre-procedural counseling and individualized pain management strategies.

From a health economics perspective, the comparative value of TPBx and TRBx remains context-dependent, with cost-effectiveness conclusions varying markedly by healthcare setting, analytic perspective, follow-up duration and so on. The TRANSLATE trial found TPBx to be more costly (£1,062 vs. £917) with no short-term Quality-Adjusted Life Year (QALY) gain (65). In contrast, the PREVENT trial reached different conclusions, suggesting that when accounting for the costs of treating complications, TPBx may be less costly overall than TRBx (66). Australian real-world data provide further insight, showing that while baseline TPBx costs declined with operator experience, integrating TPBx with MRI-based triage yielded significant savings (AUD 783 per patient) (67). These findings underscore that the economic viability of a biopsy technique depends not only on the procedure itself but also on accompanying strategies such as patient selection and institutional learning curves.

### 6.4 Grid-based or freehand transperineal biopsy

Transperineal prostate biopsy is performed via either grid-based or freehand techniques. Freehand transperineal biopsy has gradually gained favor due to its outpatient feasibility under local anesthesia and favorable safety profile. csPCa detection rates with freehand biopsy are comparable to those of grid-based mapping, while infectious complications remain near zero and pain scores are low (68–70). Crucially, freehand biopsy reduces post-procedural urinary retention compared with grid-based approaches (1% vs. 10%), while paradoxically yielding a higher mean number of csPCa-positive cores per case (71).

The freehand technique confers distinct advantages in anatomically challenging zones where grid templates restrict needle angulation. Unconstrained needle mobility enables steep trajectories into the anterior fibromuscular stroma and apical regions, thereby accessing areas that grid-based approaches often sample suboptimally or miss entirely (70). In the setting of MRI-targeted biopsy, this flexibility translates into consistent csPCa detection rates across all prostatic zones (72). When systematic sampling is employed without MRI guidance, zonal contributions diverge markedly: Urkmez et al. reported that freehand biopsy yielded uniquely high rates of csPCa uniquely involving the anterior sector (22% anteromedial, 51% anterolateral), figures that notably surpass those historically observed with grid-based mapping. Furthermore, extended systematic templates reveal that the basal sector contributes minimally to independent csPCa detection—only a single case (1.5%) was confined exclusively to this region (68)—underscoring the disproportionate diagnostic importance of the anterior and apical zones. Collectively, these findings demonstrate that freehand TPBx, by virtue of its unrestricted needle trajectory, achieves superior sampling of the anterior and apical prostate compared with grid-based templates, thereby enhancing csPCa detection in regions where conventional approaches are most constrained.

AI is increasingly deployed to address the inherent variability of cognitive freehand navigation. AI-driven auto-contouring accelerates fusion imaging and narrows the expert-novice performance gap (69), while sensorless deep-learning probe tracking achieves sub-2.5 mm real-time registration accuracy without external hardware (73). By enhancing both targeting precision and workflow efficiency, AI integration promises to standardize freehand TP biopsy across varying operator experience levels. Collectively, these innovations position AI-augmented freehand transperineal biopsy as a paradigm that harmonizes diagnostic accuracy, procedural safety, and resource-conscious outpatient practice.

## 7 The role of artificial intelligence and emerging technologies

In recent years, the rapid advancement of AI has driven the intellectualization of prostate cancer diagnosis across the entire workflow, from imaging to pathology. Numerous AI models have been developed (74), with multimodal AI (MMAI) models now incorporated into clinical EAU guidelines, integrating pathological images with clinical data to predict prostate cancer-specific mortality and overall survival (75). AI has also been proposed as a tool to offer potential cost efficiencies, with some modeling studies suggesting a reduction of up to 80% in the number of biopsy cores requiring pathological evaluation. However, these estimates are primarily derived from retrospective or hypothetical analyses and require rigorous prospective economic validation before they can be translated into routine practice (76). During biopsy, AI aids target selection and puncture guidance, minimizing unnecessary cores while maintaining detection of csPCa. In post-biopsy pathological specimen analysis, AI not only distinguishes benign from malignant tissue but also improves GG grading. By advancing precision and individualization in prostate cancer management, this section focuses on the current applications of these technologies in prostate biopsy, summarizing high-quality clinical evidence and discussing future directions.

### 7.1 The role of AI during biopsy

During biopsy, deep learning models enable automatic real-time segmentation of prostate zones on TRUS images, addressing the operational inefficiencies, labor intensity, and costs associated with cog-biopsy (77). ProCUSNet is a nnU-Net-based model that segments the prostate and csPCa on 3D-reconstructed ultrasound images. Even conventional ultrasound images reconstructed from screenshots, when processed through AI training, reliably localized csPCa (78). However, its current performance has been demonstrated primarily on retrospective internal datasets or reconstructed screenshots, and it has not yet undergone robust external validation across diverse ultrasound platforms or patient populations. This AI-driven approach has the potential to function as an intelligent adjunct to SBx, offering MRI-level targeting capability in settings where MRI is unavailable or impractical.

By contrast, a recent prospective, multicenter RCT compared AI-assisted cognitive biopsy with conventional cognitive biopsy. In this independent clinical validation cohort, the AI-assisted approach significantly improved the detection rate of csPCa (58.6% vs. 46.6%) while reducing the detection of cisPCa (6.3% vs. 11.1%) (79). By integrating real-time zonal segmentation with suspected lesion localization, such models effectively transform a conventional ultrasound platform into a low-cost, accessible targeting tool. This “intelligent cognitive biopsy” paradigm may prove particularly valuable in low- and middle-income regions where MRI availability remains limited. Further prospective validation across diverse populations and platforms will be essential to establish generalizability and facilitate widespread clinical adoption.

### 7.2 Research progress of assistive robots

Robotic-assisted technology has advanced prostate cancer biopsy by enhancing precision and automation. However, many of these innovations remain at the preclinical, algorithmic proof-of-concept, or retrospective feasibility stage. For instance, the TransVFS technology can improve needle-tissue interaction force estimation, enabling safer and more accurate needle placement even with low-resolution ultrasound or indistinct prostate boundaries (80). These findings, however, derive from phantom or *ex vivo* studies and have not yet been validated in live patient cohorts. Concurrently, to address the high computational cost of Transformers and the limited global modeling capability of CNNs, the MixUNETR architecture has been proposed. This hybrid deep learning model, validated on retrospective MRI datasets (internal validation), achieves accurate segmentation of the peripheral and transition zones of the prostate (81). To translate these advances into clinical application, researchers developed ProBot, a robotic-ultrasound probe for both transrectal and transperineal prostate biopsies. Its two-degree-of-freedom side-firing design minimizes prostate deformation and fusion errors while supporting MRI-ultrasound fusion and multiple biopsy routes, requiring large-scale prospective clinical cohorts (82). These foundational advances collectively drive the intelligent evolution of precision PCa diagnosis, laying essential groundwork for enhanced perceptual accuracy and operational autonomy in next-generation robotic-assisted systems.

At the molecular imaging level, another prospective single-center cohort study evaluated the safety and diagnostic performance of ^68^Ga-PSMA-PET/CT-guided robotic-assisted prostate biopsy. The findings demonstrate that this approach offers a high safety profile and excellent diagnostic yield, particularly in patients with prior negative biopsies and anterior PSMA-positive lesions, achieving a sensitivity of 91% (83). Together, these integrated systems demonstrate how advancements in AI-driven perception and robotic hardware are translating into clinically meaningful improvements in diagnostic accuracy. By addressing both technological foundations and clinical integration, these innovations collectively pave the way for more precise, personalized, and accessible prostate cancer diagnosis.

### 7.3 The role of AI in biopsy pathology

AI is increasingly integrated into prostate cancer pathology, extending beyond traditional diagnosis. One study demonstrated that an AI algorithm using only H&E-stained images effectively predicts metastasis risk after radical prostatectomy, retaining predictive value even with limited tissue from core biopsies, and performance improves when combined with clinical variables, positioning AI as a low-cost, accessible risk stratification tool (84). In diagnostic workflows, AI systems correctly flagged 55% of equivocal cases that would otherwise require immunohistochemistry, with an error rate of just 1.4%. As a standalone detector, the model achieved an AUC of 98.5%, with 95.0% sensitivity and 97.8% specificity, helping identify small cancers initially missed and optimizing pathology workflows (85).

Emerging technologies also enable real-time intraoperative diagnosis. Stimulated Raman Histology (SRH), a label-free imaging technique, combined with convolutional neural networks, was evaluated in a prospective validation cohort and achieved 96.5% accuracy for prostate cancer detection (sensitivity 96.3%, specificity 96.6%). Scanning time was reduced to 2–2.75 minutes, and as little as one minute for cancer-containing regions. While these findings are promising, they were derived from a single-center prospective cohort; external validation in independent patient populations and across multiple institutions are required to confirm generalizability (86). Together, these advances underscore AI's expanding role across the diagnostic continuum—from detection and risk stratification to intraoperative guidance.

## 8 Discussion

### 8.1 From standardization to personalization: weighing the evidence from biopsy strategies

The preceding contents have chronicled a decisive paradigm shift in prostate cancer diagnosis, moving from a one-size-fits-all systematic sextant approach to nuanced, patient-specific methods. The binary choice between SBx and TBx has been supplanted by the recognition that CBx optimizes detection but carries an inherent risk of overdiagnosis. The VISION meta-analysis and the PRECISE trial 2-year follow-up robustly demonstrated that while TBx improves csPCa detection efficiency and reduces cisPCa overdetection, the PROBASE trial serves as a critical counterweight, revealing that SBx missed fewer csPCa cases in younger men (6, 9). This tension underscores that the optimal strategy is not a universal constant but a variable contingent on prostate-specific conditions.

The evolution toward extended targeted strategies—PBx, uni-SBx, TSx—further refines this personalization. These approaches represent a conscious effort to de-escalate sampling in low-yield regions while intensifying scrutiny of the index lesion. The non-inferiority of PBx regarding csPCa detection, coupled with lower complication rates as shown in the PERI-PRO RCT, suggests a viable path to minimizing patient morbidity (21). However, an extension of this thinking invites a crucial, unanswered question: Are we ready to accept the trade-off inherent in uni-SBx? The meta-analytic data confirms that contralateral systematic biopsy contributes to only ∼1.5% of exclusive csPCa diagnoses (24). Yet, in an era of patient-centered care, this 1.5% represents a missed opportunity for cure in a patient who may present with metastatic disease years later. The future of personalized biopsy may hinge less on the absolute avoidance of missed diagnoses and more on transparently integrating this statistical risk into shared decision-making frameworks.

### 8.2 Navigating the complexities of biopsy guidance and route selection

The technical modality of guidance and the anatomical route of access represent two further axes of personalization where evidence supports contextual superiority rather than a single gold standard. The comparative equipoise between in-bore TBx, fus-TBx, and cog-TBx suggests that institutional expertise and lesion location should drive technique selection (32). Notably, the emergence of micro-ultrasound as a non-inferior alternative to mpMRI-guided biopsy in the OPTIMUM trial challenges the hegemony of MRI, potentially democratizing access to precision imaging in resource-constrained settings (46).

The TPBx versus TRBx debate epitomizes the clinical trade-offs. The large-scale RCTs in Table 1 generally support comparable overall csPCa detection between TPBx and TRBx. Subgroup and anatomical analyses suggest that TPBx is non-inferior, and may be superior in some subgroups, for detecting anterior and apical csPCa, while nearly eliminating sepsis risk (54, 59, 60, 62). Evidence regarding infectious complications is more heterogeneous across trials, although several studies suggest a potential reduction in infectious events with TPBx (54, 55). Yet, the cost-effectiveness analysis from TRANSLATE and the nuanced pain perception data from PREVENT remind us that “non-inferiority” does not equate to “universal superiority” (65). A forward-looking perspective suggests that the future of route selection may be dictated by the fusion of AI-driven needle tracking with freehand TPBx. By eliminating the constraints of a fixed grid, freehand TPBx enhances access to the anterior fibromuscular stroma and facilitates steep needle angulation. This approach, when coupled with AI-assisted contouring and real-time navigation, could represent the next leap in procedural efficiency—transforming the biopsy from a blind sampling exercise into a precision-guided, outpatient procedure.

### 8.3 The promise and perils of artificial intelligence

AI is poised to disrupt the prostate cancer diagnostic continuum, from real-time ultrasound segmentation to digital pathology interpretation. AI-assisted cognitive biopsy has already demonstrated superiority over conventional cognition, improving csPCa detection rates by effectively augmenting the operator's spatial reasoning (79). Furthermore, AI's potential to reduce pathology workload by up to 80% and accurately predict metastasis from H&E slides represents a paradigm shift in resource allocation and risk stratification (76, 84).

However, the translation of these proof-of-concept successes into widespread clinical practice remains fraught with challenges. The current literature is dominated by single-institution, retrospective studies with proprietary algorithms that lack rigorous external validation. An essential extension of this discussion must address the specter of algorithmic bias. AI models trained predominantly on homogeneous, high-income population datasets risk perpetuating and amplifying health disparities in prostate cancer outcomes for Black men and other underserved groups. Moreover, the medicolegal and regulatory framework for “intelligent cognitive biopsy” remains undefined. Who is liable when an AI-driven segmentation error leads to a missed anterior tumor? The path forward requires not just better algorithms, but the implementation science to validate these tools across diverse populations and workflows, ensuring that AI serves as an equity-enhancing adjunct rather than a new vector for diagnostic disparity.

### 8.4 Conclusion: embracing complexity in the era of precision medicine

This narrative review has traversed the evolution from traditional methods to AI-augmented freehand transperineal targeting, revealing a field in dynamic flux. The central thesis—that we are witnessing a paradigm shift from standardization to personalization—is supported by a robust, albeit incomplete, evidence base. We have moved beyond the false dichotomy of SBx versus TBx; the clinical imperative now is to orchestrate the available tools—mpMRI, micro-US, PSMA-PET/CT, and robotic platforms—in a manner that balances diagnostic yield with patient comfort and healthcare economics.

Yet, a significant gap remains between the aspiration of personalization and its operationalization. We possess the moderators (PI-RADS, PSAD, volume and so on) but lack the validated, dynamic algorithms to weigh them in real time. The next frontier of research must pivot from comparative effectiveness trials of fixed protocols toward adaptive platform trials that evaluate strategy selection based on individual patient vectors. Until such frameworks mature, the prudent clinician will navigate this complexity not by seeking a universal best method, but by fostering an informed dialogue that calibrates the intensity of the diagnostic search to the unique biological and personal context of the patient at hand. The defining transformation of modern prostate cancer diagnosis is not the abandonment of standardization, but its thoughtful subjugation to the principles of precision medicine.

## 9 Critical review

Despite substantial advances in prostate biopsy techniques, the field has not yet achieved true precision biopsy. Most contemporary studies still compare predefined protocols, such as systematic, targeted, combined, perilesional, or unilateral biopsy, rather than prospectively determining which strategy is optimal for an individual patient. Although factors such as PI-RADS score, PSA density, prostate volume, lesion location, and prior biopsy history may modify diagnostic yield, current evidence largely derives from subgroup analyses rather than validated strategy-selection models. Thus, current “personalization” remains primarily a refinement of fixed protocols rather than a truly individualized diagnostic framework.

A second limitation is the continued reliance on csPCa detection as the principal endpoint. Higher detection does not necessarily translate into greater clinical benefit, because additional cancer detection may be accompanied by increased overdiagnosis, biopsy burden, complications, and downstream treatment. Conversely, biopsy de-escalation inevitably introduces a risk of missed csPCa, and population-level reductions in sampling may obscure the clinical importance of individual missed cases. Future studies should therefore move beyond simple detection comparisons and evaluate net clinical benefit, incorporating missed csPCa, overdiagnosis, complications, patient-reported outcomes, costs, and downstream treatment consequences. Importantly, missed cancers should be characterized according to grade, volume, and clinical significance rather than treated as a uniform endpoint.

The heterogeneity of existing studies also limits direct comparison between biopsy strategies. MRI quality and interpretation, operator experience, fusion platforms, biopsy templates, number of cores, definitions of csPCa and complications, and peri-procedural management may all influence outcomes. This is particularly relevant when interpreting apparent advantages of TPBx, PBx, or emerging technologies. Future comparative studies should therefore standardize clinically relevant endpoints and explicitly account for operator and institutional effects. Non-inferiority should also be interpreted cautiously: demonstrating statistical non-inferiority for csPCa detection does not establish superiority in overall clinical value, particularly when the proposed benefit lies in reduced sampling, morbidity, or cost.

The next step should therefore be a shift from repeated head-to-head comparisons toward “strategy-selection research”. Rather than asking which biopsy technique is universally superior, future trials should investigate whether clinical, imaging, anatomical, and patient-level factors can be integrated to determine the appropriate biopsy route, targeting method, sampling intensity, and need for systematic cores. Adaptive or risk-stratified trial designs may be particularly valuable. For AI, the key challenge is similarly no longer demonstrating technical diagnostic performance, but establishing external validity, clinical utility, and outcome improvement across diverse populations and practice settings. Ultimately, precision biopsy should aim not simply to detect more cancer, but to identify clinically consequential cancer while minimizing unnecessary biopsy, overdiagnosis, and procedural harm.
